# On Varioloid and Varicella

**Published:** 1856-04

**Authors:** 


### *On Varioloid and Varicella.* By Professor TROUSSEAU.

(Medical Times and Gazette, Sep. 1, 1855.)

Many practitioners of high scientific repute believe that the same relationship prevails between varicella and varioloid as between this last and variola. This it is impossible to admit. If we bring an individual having genuine vaccine scars in contact with a smallpox patient, he may take a varioloid, and, while suffering from this, he may communicate a true variola to a subject who has neither been vaccinated nor had the smallpox. If we take the pus from a varioloid patient, and inoculate a healthy person, as has been done in epidemics when vaccine lymph has run short, we produce the legitimate smallpox. These are so many proofs of the identity of the two affections. It is not thus with varicella. It neither arises from contact with varioloid, or is capable of communicating true variola. We see it arise just as easily in persons who have had that disease, as in those who have been exempt from it; in the unvaccinated, and in those who have been vaccinated. M. Trousseau has seen an epidemic of varicella at the Necker Hospital, which attacked all the children, a short time after vaccination had been quite successful. This is an important question in hygiene, inasmuch as varicella, of itself, is an affection destitute of danger; and we may leave the subject of it in communication with surrounding persons, without the fear of finding a serious malady developed. The same practice, pursued in varioloid, might give rise to a mischievous development of variola.

*Varioloid.*—Thirty-five years ago, an authentic example of smallpox after vaccination was unknown, although Jenner had seen examples of this, and had indicated them; but, as there are always to be found persons more royal than the king, so there were practitioners who accorded to vaccine more than he who had discovered and propagated it had claimed for it. In 1825, a very violent epidemic of smallpox prevailed in Paris, during which individuals who had been vaccinated were attacked. M. Husson, who was one of the Vaccine Committee in 1800, and one of the most ardent promoters of vaccination, contested the validity of these cases; and so extraordinary was the circumstance thought to be, that whenever a varioloid patient arrived at the hospital, the bells were loudly rung, in order to call as great a number of practitioners together as possible for the verification of the fact. An epidemic at Edinburgh, and two at Marseilles, multiplied examples. The attention of governments became aroused, and especially in Germany, where re-vaccination has been rendered obligatory. At the present day, there is no hospital in which we may not see persons having the vaccine scars the subject of variola, and even dying of it. It may occur even as early as the second or third year after vaccination; and M. Trosseau has seen an infant at the Necker Hospital take a genuine variola six weeks after a successful vaccination. A mother and her three children also took it soon after vaccination, and in the woman, who died, it was confluent.

At its onset varioloid differs in nowise from variola. Fever arises and continues until the eruption appears. We see, however, more frequently supervene a scarlatini form or petechial eruption, but it does not influence the prognosis unfavorably, as in variola. The eruption does not differ from that of variola, until the eighth day; but at the eighth day from the commencement, or the fourth from the eruption, in place of tumefaction and inflamed areola supervening, we find the integuments become pale and flaccied. The pustules do not become larger, remain acuminated, and umbilicate but little. They dry without bursting, become rugous, and pass into the "horny" condition. Those of the limbs, in place of acquiring a size three or four times as large as those of the face, do not increase, and cornify in the same manner. By the tenth day the eruption is dry. In more serious forms, when the varioloid, as sometimes happens, is confluent, there is sometimes secondary fever; but at the tenth day, the tumefaction stops short, without any accident supervening, while in variola its doing so would be of fatal augury. The whole terminates with a rapid desquamation, although marks may remain, especially in persons with delicate skins.

*Varicella.*—When a child is brought to the Necker with varicella, the date of its admission is noted, and sixteen or seventeen days later, other children always exhibit the disease. If, on the contrary, it had been a smallpox case, other cases would have been observed from nine to eleven days afterwards—showing that the period of incubation is very different in the two affections. A child, in good health, whether vaccinated or not, whether having had variola or not, becomes suddenly the subject of a sharp attack of fever, there being present neither vomiting nor lumbar pain. The next day, or sometimes even the same day, fifteen or twenty red points are observed upon the skin, and some hours later the epidermis is raised. Twenty-four hours after the appearance of the red points, we observe bullæ or phlyctænæ, quite rounded in form, and transparent, as if they contained water. They resemble suadamina, magnified from ten to fifteen times. In variola and varioloid the eruption never assumes this bullar form. In those diseases, too, the fever and the eruption continue until the latter is completed. In varicella the phenomena take place successively. There is a day of complete apyrexia, the fever comes on during the night, and next day we find from thirty to forty points of eruption. The same takes place during the next twenty-four hours, and so on for four or five days, so that we have four or five successive eruptions. Twelve hours after the appearance of the eruption there is a limpid bulla formed, and forty-eight hours after the liquid has become lactescent, which is never observed in any form of variola. In variola discreta the eruption is of a very regular, rounded form, like a drop of wax dried upon the skin; but after two or three days the bullæ of varicella become unequal, irregular, and puckered, but never offer any appearance of umbilication. When pus begins to form in the phlyctænæ, a livid red, inflammatory areola is produced, larger in size than the variolous areola. When the pustule bursts it leaves a dark brown scab, having nothing in common in appearance with the yellow scab in variola, but much resembling that of echthyma. From twelve to fifteen days are required for the complete evolution of a variolous pustule, while four, or at most five, days suffice in varicella. So little dangerous is this affection, that M. Trousseau knows of no example of its having terminated fatally. Still, in some children, who manifest the purulent diathesis, it is followed by successive eruptions of pemphigus, which terminate by exhausting the patient and causing death. But these deaths cannot be imputed to the varicella itself.

Thus, then, variola and varioloid are identical; while varicella is distinguished from these by the differences in its period of incubation and febrile paroxysms, by the form and duration of its eruption, and by the absence of danger.—*Ranking's Abstract.*

